# Exploration of intramolecular split G-quadruplex and its analytical applications

**DOI:** 10.1093/nar/gkz749

**Published:** 2019-08-31

**Authors:** Mengmeng Lv, Yuchun Guo, Jiangtao Ren, Erkang Wang

**Affiliations:** 1 State Key Laboratory of Electroanalytical Chemistry, Changchun Institute of Applied Chemistry, Chinese Academy of Sciences, Changchun, Jilin 130022, China; 2 University of Chinese Academy of Sciences, Beijing 100049, China; 3 College of Chemistry, Jilin University, Changchun, Jilin 130012, China

## Abstract

Distinct from intermolecular split G-quadruplex (Inter-SG), intramolecular split G-quadruplex (Intra-SG) which could be generated in a DNA spacer-inserted G-quadruplex strand has not been systematically explored. Not only is it essential for the purpose of simplicity of DNA-based bioanalytical applications, but also it will give us hints how to design split G-quadruplex-based system. Herein, comprehensive information is provided about influences of spacer length and split mode on the formation of Intra-SG, how to adjust its thermodynamic stability, and selection of optimal Intra-SG for bioanalysis. For instances, non-classical Intra-SG (e.g. 2:10, 4:8 and 5:7) displays lower stability than classical split strands (3:9, 6:6 and 9:3), which is closely related to integrity of consecutive guanine tract; as compared to regular Intra-SG structures, single-thymine capped ones have reduced melting temperature, providing an effective approach to adjustment of stability. It is believed that the disclosed rules in this study will contribute to the effective application of split G-quadruplex in the field of DNA technology in the future.

## INTRODUCTION

G-quadruplex DNA as one type of significant functional nucleic acid, has been studied widely and introduced into various applications, such as biosensors and logic systems (1). It has a four-stand structure and consists of two or more G-tetrads generated by formation of eight hydrogen bonds among four guanines. At least four consecutive guanine (G_*n*_) repeats exist in a unimolecular G-quadruplex structure, e.g. T30695 sequence d (G_3_T)_4_ (2) and C-Myc promoter sequence d(TGAG_3_TG_4_AG_3_TG_4_A_2_) (3). In the past decade, G-quadruplex-mediated non-covalent signaling methods were reported extensively based on peroxidase-mimicking activity of G-quadruplex/hemin and G-quadruplex-specific luminescent enhancement of organic dyes (4–6).

Since split G-quadruplex strategy was for the first time proposed for discrimination of single nucleotide polymorphisms (7), it has been optimized and investigated comprehensively (8–10). Researchers split a unimolecular G-quadruplex strand into two halves, and each of them was flanked with the sequences complementary to target DNA. Hybridization between target DNA and the recognition segments in binary probes promotes G-rich sequences in proximity to fold into G-quadruplex structure, resulting in colorimetric or fluorescent signals. As a intramolecular G-quadruplex sequence contains four G_*n*_ repeats, traditional binary split G-quadruplex probes were produced by dissections at positions, resulting in one, two, or three runs of guanine residues in each strand, i.e. split mode 2:2, 1:3 and 3:1 (9). Later, our group took guanine base number into account, instead of G_*n*_ tract, and obtained more split G-quadruplex probes, e.g. 8:4 binary probes which exhibited the best analytical performance (10).

**Scheme 1.**
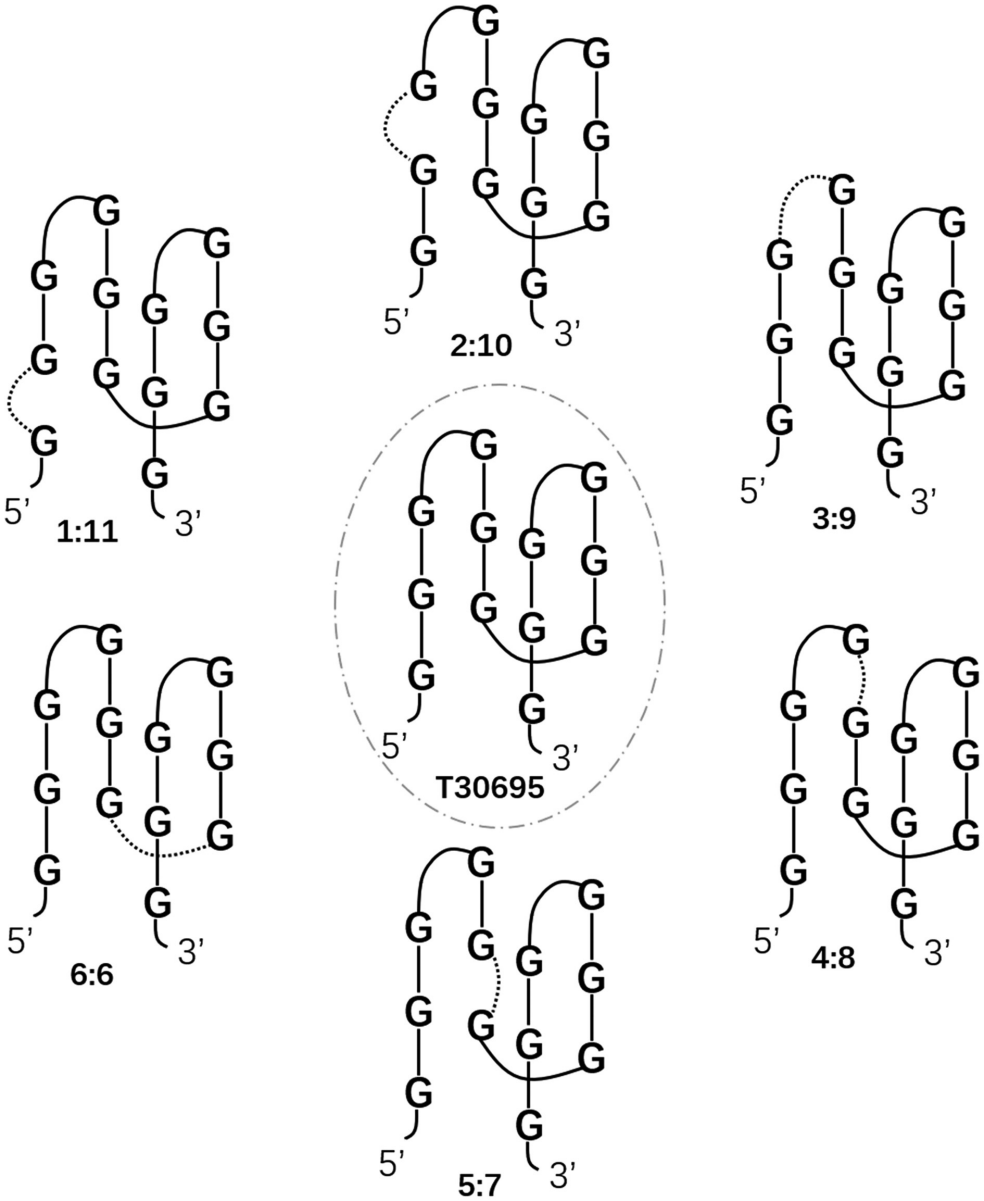
Six representative intramolecular split G-quadruplex (Intra-SG) structures derived from T30695.

It is obvious that the refolded G-quadruplex is an intermolecular motif stabilized by double-stranded DNA. We are curious that, if we insert certain length of DNA spacer in the different sites of intact G-quadruplex sequence, what it will happen. In other words, a G-quadruplex sequence is split into two segments which are linked with consecutive DNA bases, and thus a new G-quadruplex structure may be produced, which is nominated as intramolecular split G-quadruplex (Intra-SG, Scheme 1). There have been a few related reports on utilization of Intra-SG for illumination of logic operations and biosensing (11–13). For example, Yang group constructed various logic gates (OR, INHIBIT, AND and XOR) and combinational logic gates (INHIBIT-OR), using Intra-SG-clamped molecular beacon (MB) (12). However, none of them paid attention to the influence of spacer length and G-quadruplex split mode on the formation of Intra-SG and its properties, e.g. Intra-SG-stimulated fluorescence change.

Herein, the research on Intra-SG conduces to simplifying the split G-quadruplex strategy from binary probes to single probe in analytical applications based on a G-quadruplex specific fluorescent probe. Moreover, we are able to acquire more information about influence of spacer length and split mode on formation of Intra-SG and its interaction with ligand, diverse split G-quadruplex variants of distinct thermodynamic stabilities, and selection of appropriate Intra-SG for analytical purpose.

## MATERIALS AND METHODS

### Materials

All the HPLC-purified oligonucleotides (Supplementary Table S1) were obtained from Sangon Biotechnology Co., Ltd (Shanghai, China). *N*-Methyl mesoporphyrin IX (NMM) was purchased from Frontier Scientific, Inc. (Logan, Utah, USA). Potassium chloride and magnesium chloride were purchased from Sinopharm Group Chemical Reagent Co., Ltd (Shanghai, China). Oligonucleotides dissolved in Tris-HCl buffer (25 mmol/l, pH 8.0 or 7.0) were quantified using UV–Vis absorption spectroscopy and stored at –20°C. These oligos were heated at 95°C for 3 min and gradually cooled to room temperature before use. NMM stock solutions (5 mmol/l) in DMSO were stored in the dark at –20°C and diluted with ultrapure deionized water. Other chemicals were used as received without further purification. Ultrapure deionized water was used throughout. Most of samples were prepared in Tris–HCl buffer at pH 8.0, but the experiments for triplex formation were implemented at pH 7.0.

### Circular dichroism measurements

**Figure 1.**
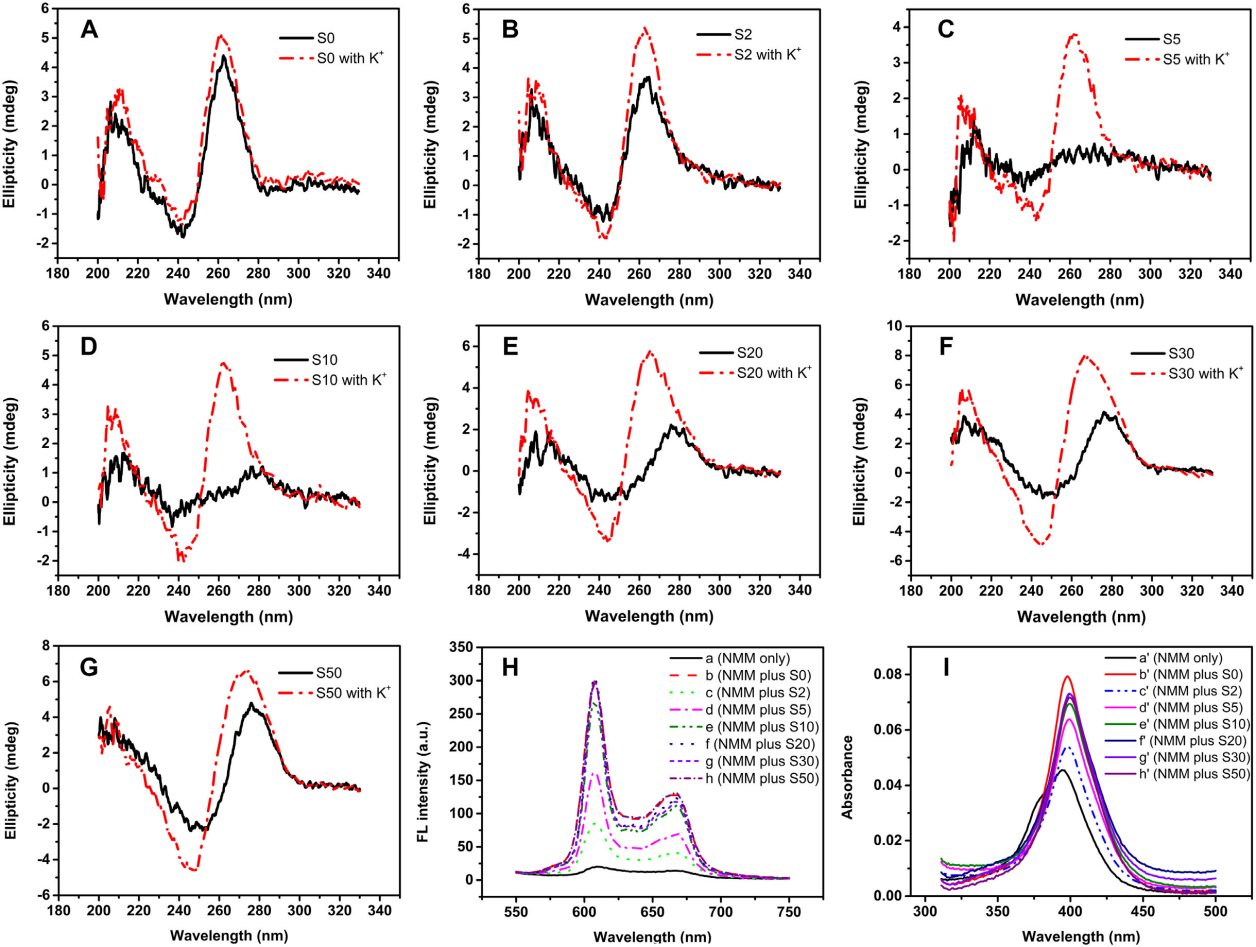
(**A**–**G**) CD spectra of Intra-SG strands with different spacer lengths without or with 300 mM K^+^. The final concentration of indicated Intra-SG strand was 7 μM. (H) Fluorescence spectra of NMM with distinct Intra-SG strands (b–h) in the presence of 300 mM K^+^. Curve a represents NMM only without any DNA strand. The final concentrations of NMM, Intra-SG strand and K^+^ in Tris–HCl buffer (25 mM, pH 8.0) were 1 μM, 300 nM and 300 mM, respectively. The excitation wavelength was 399 nm and the slits were 10/10 nm. (I) UV–vis spectra of NMM mixed with distinct Intra-SG strands (b′–h′). Curve a′ represents NMM only without any DNA strand. The final concentrations of NMM, indicated Intra-SG strand and K^+^ in Tris–HCl buffer (25 mM, pH 8.0) were 1 μM,1.5 μM and 300 mM, respectively.

Each 200 μl sample containing 7 μmol/l DNA strand and metal ions (K^+^ or Mg^2+^) of certain concentration was prepared and incubated for 1 h at 20°C before measurement. Circular dichroism (CD) spectra from 200 to 350 nm were obtained (Figures 1A–G, 3, 5B, Supplementary Figures S1A–K and S6A) on a JASCO J-820 spectropolarimeter (Tokyo, Japan) with a cuvette of 1 mm light path at 20°C. Each CD spectrum was corrected by automatically subtracting the background.

### Thermal melting experiments

**Figure 2.**
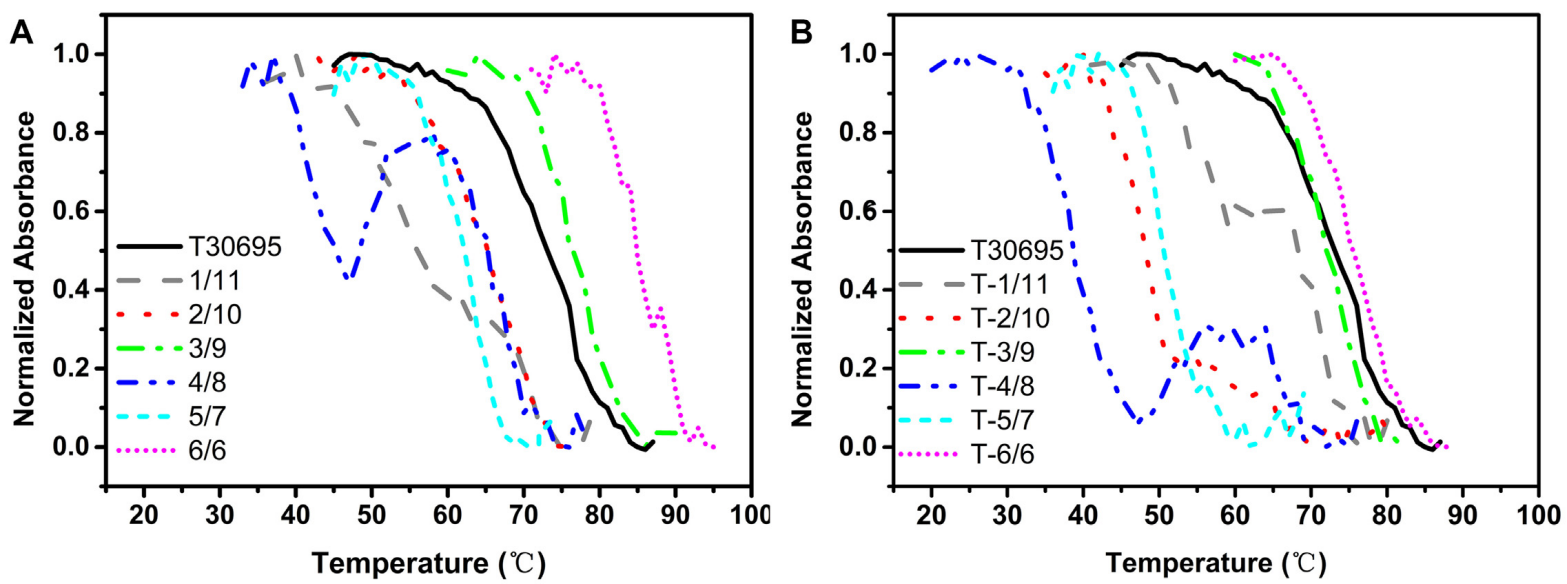
Normalized UV-melting profiles of T30695 and regular Intra-SG strands (**A**) and single-T capped Intra-SG strands (**B**) at 295 nm versus temperature, in the presence of 300 mM K^+^. The corresponding *T*_m_ values were shown in Table 1. The concentration of each DNA strand in Tris–HCl buffer (25 mM, pH 8.0) was 2 μM.

750 μl samples (Figures 2, 5C, Supplementary Figures S3, S4 and S6B) containing 2 μmol/l DNA strand and metal ions (K^+^ or Mg^2+^) of certain concentration were prepared and incubated for one hour. All the samples were covered with paraffin liquid. UV measurements were performed on a Cary 60 UV–vis spectrometer (Agilent, USA). Absorption spectra from 200 to 350 nm was collected as a function of temperature at a ramping rate of 1°C/min. UV-melting profiles were drawn at 295 nm for DNA G-quadruplex, and 260 nm for DNA duplex and triplex. Melting temperature (*T*_m_) of each sample was obtained by first derivative analysis of obtained denaturation curves in the data-processing software of Origin 9.0.

### UV analysis of DNA–NMM complex

Solutions containing 1 μmol/l NMM, 1.5 μmol/l Intra-SG strands with different spacer lengths and 300 mM K^+^ were prepared (Figure 1I), and incubated for 1 h at 20°C. The Soret band of NMM was recorded using UV–vis spectrophotometer. The binding of DNA strands to NMM was demonstrated according to the hyperchromicity of the NMM Soret band.

### Fluorometric assays

Fluorescence samples (300 μl) in Figures 1H, 4, 5D–E, Supplementary Figures S1L, S5 and S6C–D contained NMM (1 μmol/l), DNA strand (300 or 150 nmol/l) and metal ions (K^+^ or Mg^2+^) of indicated concentration. The mixtures were incubated for 1 h at 20°C prior to the quantification of NMM fluorescence. Fluorescence spectra were collected on a Cary Eclipse Fluorescence spectrophotometer (Agilent, USA), utilizing slits of 10/10 nm, a scan rate of 600 nm/min, excitation at 399 nm and emission at 608 nm.

## RESULTS AND DISCUSSION

### Intra-SG design and DNA spacer effect

A typical G-quadruplex sequence, T30695 (GGGTGGGTGGGTGGGT), was selected and could fold into G-quadruplex (14–16). By inserting a spacer in the distinct sites of T30695 sequence (Scheme 1 and Scheme 1S), eleven types of Intra-SG sequences would be produced, corresponding to split mode 1:11, 2:10, 3:9, 4:8, 5:7, 6:6, 7:5, 8:4, 9:3, 10:2 and 11:1, respectively. As a matter of fact, 3:9, 6:6 and 9:3 (namely, classical split mode 1:3, 2:2 and 3:1, respectively) were usually adopted in a number of intermolecular split G-quadruplex (Inter-SG) systems (7,9,17,18). In a previous report, it was found that 8:4 Inter-SG probes exhibited the best performance toward target detection (10). But it is unknown yet whether the rule is applied to Intra-SG probes. It is quite probable that these Intra-SG sequences possess different capabilities to fold into G-quadruplex. To compare their thermodynamic stabilities is essential, and how to adjust stability is very meaningful for their applications. In this work, a common organic dye, *N*-methyl mesoporphyrin IX (NMM), was employed as non-covalent fluorescent indicator for G-quadruplex structure, and provided readable signal of sensing systems.

Before the comprehensive investigation of split mode, how many consecutive bases as a DNA spacer should be inserted between two split segments was studied firstly in the presence of 300 mM potassium ion (K^+^). S0, S2, S5, S10, S20, S30 and S50 correspond to Intra-SG (8:4) sequences with 0, 2 5, 10, 20, 30 and 50 consecutive thymine bases at the split site, respectively (Supplementary Table S1). First of all, CD spectroscopy was utilized to directly confirm G-quadruplex structures (Figure 1). Both curves in Figure 1A exhibit a positive peak at 264 nm and a negative peak around 245 nm, illustrating a characterized G-quadruplex structure can be generated from T30695 strand, regardless of whether K^+^ is present (19). Unexpectedly, similar to T30695, S2 folded into a G-quadruplex even without K^+^ (Figure 1B). As for S5, S10, S20, S30 and S50, the peak at 264 nm was observed only after addition of K^+^ (Figure 1C–G). The data elucidates that G-quadruplex could be yielded in the Intra-SG sequences, even if the spacer length attains 50 bases. As reported previously (20), T30695 (S0) enhanced the fluorescence of NMM apparently (curve a and b, Figure 1H), because of complexation between G-quadruplex structures and NMM. Other DNA strands did induce the enhancement but with different degrees (curve c–h, Figure 1H). High fluorescence was observed for S20, S30 and S50, similar to T30695. But relatively weak fluorescence for strands S2 and S5 (curves c and d, Figure 1H) illustrates that short spacer is detrimental to interaction between the strands and NMM, which was verified further by UV–vis absorption spectroscopy. Absorption spectra of NMM mixed with DNAs were collected and Soret band was monitored (Figure 1I), which could provide the clues about what happened to NMM (21). Red shifts and hyperchromicity of the Soret band were observed for all these DNA stands, indicating the outside binding mode of the NMM to these DNAs. Smallest hyperchromicity for S2 (curve c' in Figure 1I) discloses that short inserted spacer interferes with the interaction between NMM and G-quadruplex, thereby resulting in low fluorescent emission. To eliminate the adverse effect of spacer, 20-base spacer was adopted for the following investigation of split mode.

### Exploration of Intra-SG structures of different thermodynamic stabilities

On the basis of number of guanine bases in each split segment, eleven types of split modes were designed, leading to eleven Intra-SG sequences, 1/11, 2/10, 3/9, 4/8, 5/7, 6/6, 7/5, 8/4, 9/3, 10/2 and 11/1, with a DNA spacer of 20 bases (Supplementary Table S1). Analogous to S20, the injection of K^+^ into solutions containing the Intra-SG strands of distinct split modes triggered intensive ellipticity increase around 264 nm (Supplementary Figure S1A–K), which testified G-quadruplex formation in the presence of K^+^. No matter whether K^+^ was present, T30695 caused intensive fluorescence enhancement of NMM, as shown in Supplementary Figure S1L. Different from T30695, weak emission was observed for NMM mixed with Intra-SG DNAs, until potassium (300 mM) was added. The property of K^+^-promoted fluorescence enhancement was utilized for non-covalent fluorescent detection of potassium in solution (discussed below). It has been reported that Inter-SG structures are hardly formed without assistance of duplex, which suggests that G-quadruplexes formed from these Intra-SG sequences are intramolecular complexes. The speculation was verified by native PAGE (Supplementary Figure S2). One may observe that a new bright band is observed in all split sequences upon addition of K^+^. Therefore, formation of intramolecular complexes can be confirmed, as judged by migration markers including two single-stranded DNAs (M16 and M36) and one biomolecular G-quadruplex (AGRO100). Consequently, Intra-SG-clamped hairpin-like structures could be yileded. Considering the similarity of splitting T30695 from 5′ and 3′ terminus, we mainly show results on Intra-SG strands of split mode 1:11, 2:10, 3:9, 4:8, 5:7, 6:6, herein.

Intra-SG-formed molecular beacons could provide a simple and non-covalent platform for signal transduction. The possible distinction of thermodynamic stabilities of these Intra-SG correlates with their effective application, which has not been systematically investigated before. We implemented UV-melting experiments for T30695 and six Intra-SG DNAs. The denaturation of G-quadruplex leads to hypochromism at 295 nm (22–24). Hence, UV-melting curves of Intra-SG at 295 nm were collected (Figure 2A) and melting temperature (*T*_m_) was calculated (Table 1). Analogous to T30695, apparent denaturation curves of G-quadruplex structure were obtained for all Intra-SG sequences, in the temperature range from 30 to 85°C, and reveal that Intra-SG structures of different split modes have distinct capabilities of folding into G-quadruplex. Symmetrically split sequence 6/6 displays the highest thermodynamic stability, whose *T*_m_ (85°C) is even higher than T30695 (*T*_m_, 76°C). In addition, two melting transitions occurred for 1/11 and 4/8, suggesting the existence of a stable residual structure (25). Moreover, classical split modes (3:9 and 6:6) obviously exhibit higher thermodynamic stability than non-classical split modes (1:11 2:10, 4:8 and 5:7), which indicates that integrity of G_*n*_ tract affects the stability of split G-quadruplex evidently.

**Table 1.** *T*_m_ values of DNA strands in the presence of 300 mM K^+^, obtained by first derivative analysis of UV-melting profiles in the Figure 2 and Supplementary Figure S3

**Oligo**	1/11	2/10	3/9	4/8	5/7	6/6	T30695
*T*_m_ (°C)	51/71	66	79	41/69	63	85	76
**Oligo**	T-1/11	T-2/10	T-3/9	T-4/8	T-5/7	T-6/6	T1/CT1
*T*_m_ (°C)	53/71	49/65	73	40/66	51	74	56

**Figure 3.**
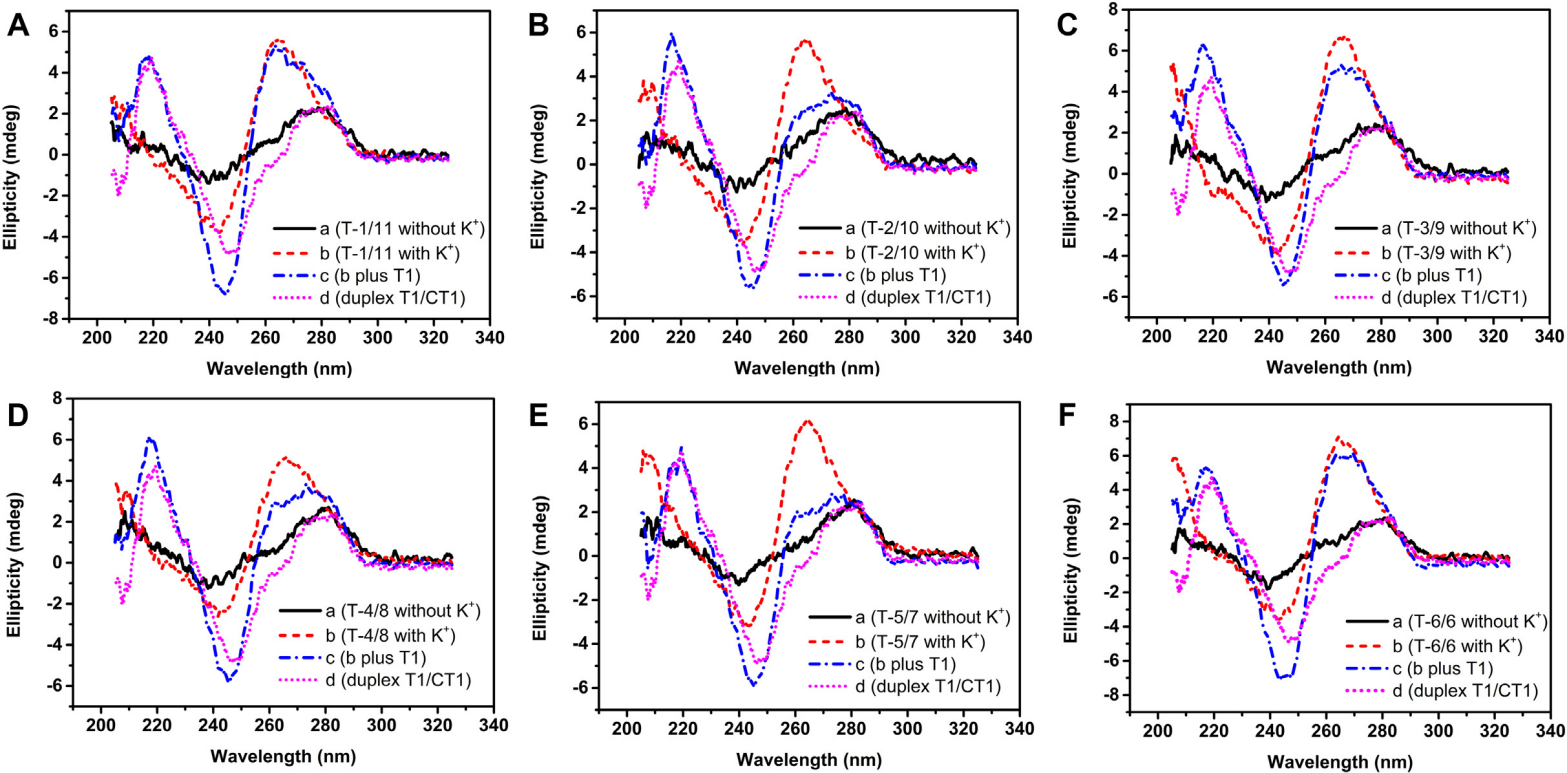
CD spectra of single-T capped Intra-SG strands without (**A**) or with (**B**) 300 mM K^+^. Curve c represents the Intra-SG structures hybridized with T1 in the presence of 300 mM K^+^. Curve d represents duplex formed between T1 and CT1. The concentration of each DNA strand in Tris–HCl buffer (25 mM, pH 8.0) was 7 μM.

Via hybridization between the spacer (loop part) and other DNA elements, Intra-SG could report the presence of different inputs. It is understandable that appropriate relative stabilities between Intra-SG and hybridized structure (e.g. duplex) are critical to feasibility of molecular recognition. To adjust stabilities of these Intra-SG and further broaden their applicable scope, extra single thymine base was added at 5′ terminus of regular Intra-SG sequences, which may interfere with structural stability of split G-quadruplex. Consequently, new Intra-SG sequences were obtained, T-1/11, T-2/10, T-3/9, T-4/8, T-5/7 and T-6/6. Characteristic CD peaks at 264 nm with K^+^ were also obtained for these single-T capped Intra-SG strands (curve b, Figure 3), confirming generation of G-quadruplex structure. The denaturation curves and *T*_m_ values are shown in Figure 2B and Table 1. In contrast to regular Intra-SG sequences, decreased thermodynamic stability was observed for these Intra-SG structures with a single-T overhang. For example, the *T*_m_ of T-6/6 is 74°C which is 11 degrees lower than 6/6. In contrast to split mode 3:9, 5:7 and 6:6, two *T*_m_ values were obtained for 1:11, 2:10 and 4:8, corresponding to two melting transitions. The phenomena suggest existence of stable residual structures which are probably derived from structural defects, i.e. damaged integrity of G_*n*_. And which *T*_m_ is decisive to the intactness of Intra-SG structure could be verified by hybridization tests below.

**Figure 4.**
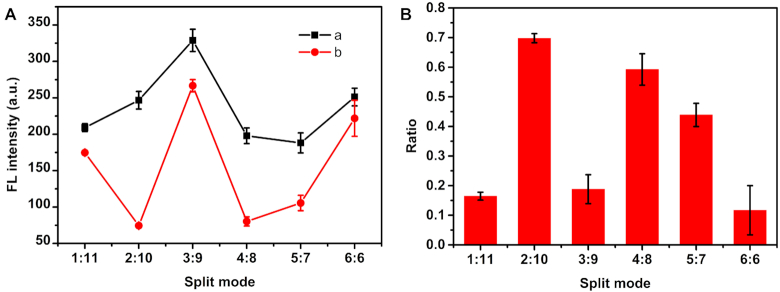
(**A**) Fluorescence intensity of NMM at 608 nm in the presence of distinct single-T capped Intra-SG strands without (a) or with (b) input of T1. (**B**) Ratio of fluorescence changes of solutions containing NMM and single-T capped Intra-SG strands, after addition of T1. The concentrations of K^+^, NMM and each DNA strand in Tris–HCl buffer (25 mM, pH 8.0) were 300 mM, 1 μM and 300 nM, respectively. The error bars indicate standard deviation of three independent measurements.

The Intra-SG structure was employed for recognizing and reporting a model target (T1) that is complementary to spacer sequence. Single-T capped Intra-SG sequences were investigated and NMM as a G-quadruplex-specific fluorescent probe was selected as the non-covalent label. Figure 4A shows G-quadruplex-specific fluorescence enhancement in the presence of single-T capped Intra-SG strands (curve a). The introduction of T1 decreased the fluorescence intensity, especially of non-classical split sequences (curve b). To be convenient for comparison, the ratio of signal change (ratio) induced by T1 was calculated according to (*F*_0_ – *F*_X_)/*F*_0_, where *F*_0_ and *F*_X_ are fluorescence intensities of solutions containing Intra-SG strands and NMM, without and with T1, respectively (Figure 4B). Maximum Ratio (69.8%) was obtained for T-2/10, which made it to be the best probe for DNA detection. Except 1:11, non-classical split modes (2:10, 4:8 and 5:7) exhibited superior detecting performance in comparison with traditional split mode 3:9 and 6:6. It is indicated that G-quadruplex-clamped molecular beacons formed from T-2/10, T-4/8 and T-5/7 could be opened by hybridization between T1 and spacer loop, leading to G-quadruplex dissociation and NMM release.

Fluorescence results are consistent with CD data in Figure 3. When T1 was introduced into solutions, two positive peaks at 217 nm and around 275 nm and an intensive negative peak at 245 nm appeared (curve c), ascribed to duplex (T1/CT1) formation. Meanwhile, the ellipticity at 264 nm of Intra-SG sequences (T-2/10, T-4/8 and T-5/7) decreased obviously, testifying the G-quadruplex unfolding. However, the remarkable features were not observed for T-3/9 and T-6/6, proving that T1 cannot open the molecular beacons clamped by G-quadruplexes of split mode 3:9 and 6:6 readily. It has to be noted that T-1/11 is an exception. Figure 3A shows that the peak corresponding to duplex occurred upon mixing of T1 with T-1/11, but CD signal at 260 nm did not change at all. It illustrates that T1 is able to be recognized by its spacer, but a G-quadruplex structure in the 11G-containing segment could still exist, which accounts for high NMM fluorescence (curve b in Figure 4A) and low fluorescence response toward T1 (Figure 4B).

The underlying reason can be interpreted by relative thermodynamic stabilities. *T*_m_ of duplexes (T1/CT1) between T1 and its complements is 56°C (Supplementary Figure S3), which is lower than the values of classical split sequences (T-3/9 and T-6/6) and higher than the first *T*_m_ values of non-classical split sequences (T-2/10, T-4/8 and T-5/7). In combination with the fluorescence and CD results, it is concluded that, classical Intra-SG-clamped molecular beacons are not likely to be opened by DNA target, due to their high stabilities; non-classical Intra-SG strands are much suitable to be designed for signaling DNA reaction, because of their moderate stabilities; first *T*_m_ value of non-classical Intra-SG represents its thermodynamic stability, and the second one is attributed to a much more stable residual structure, which is formed in only one of G-rich segments; Different from 2:10 and 4:8, the stable residual structure of 1:11 sequence is G-quadruplex, which results in low fluorescence response toward target DNA, although it does not influence recognition of target by the spacer.

To be comprehensive and convictive, *T*_m_ values of other five types of Intra-SG variants (7:5, 8:4, 9:3, 10:2 and 11:1) were also determined and are shown in the Supplementary Data (Supplementary Figure S4 and Supplementary Table S2). The common rules could be summarized: classical Intra-SG structures (split mode 3:9, 6:6 and 9:3) are more stable than non-classical Intra-SG structures; Non-classical Intra-SG structures (e.g. T-2/10) are more suitable to be introduced into nucleic acid-based reactions or sensing systems. Of note, as a unimolecular structure, Intra-SG can be formed at room temperature without addition of any extra DNA and possesses higher thermodynamic stability unsurprisingly, in contrast to respective Inter-SG structure which usually needs to be stabilized by duplex DNA and whose stability not only depends on split mode but also is decisive to the stability of assisted duplex (9,18). Intra-SG structures in this study are derived from T30695, but should be regarded as new G-quadruplexes. It is understandable that some Intra-SG structures display higher stability even than original T30695 G-quadruplex.

### Applications in biosensing systems

**Figure 5.**
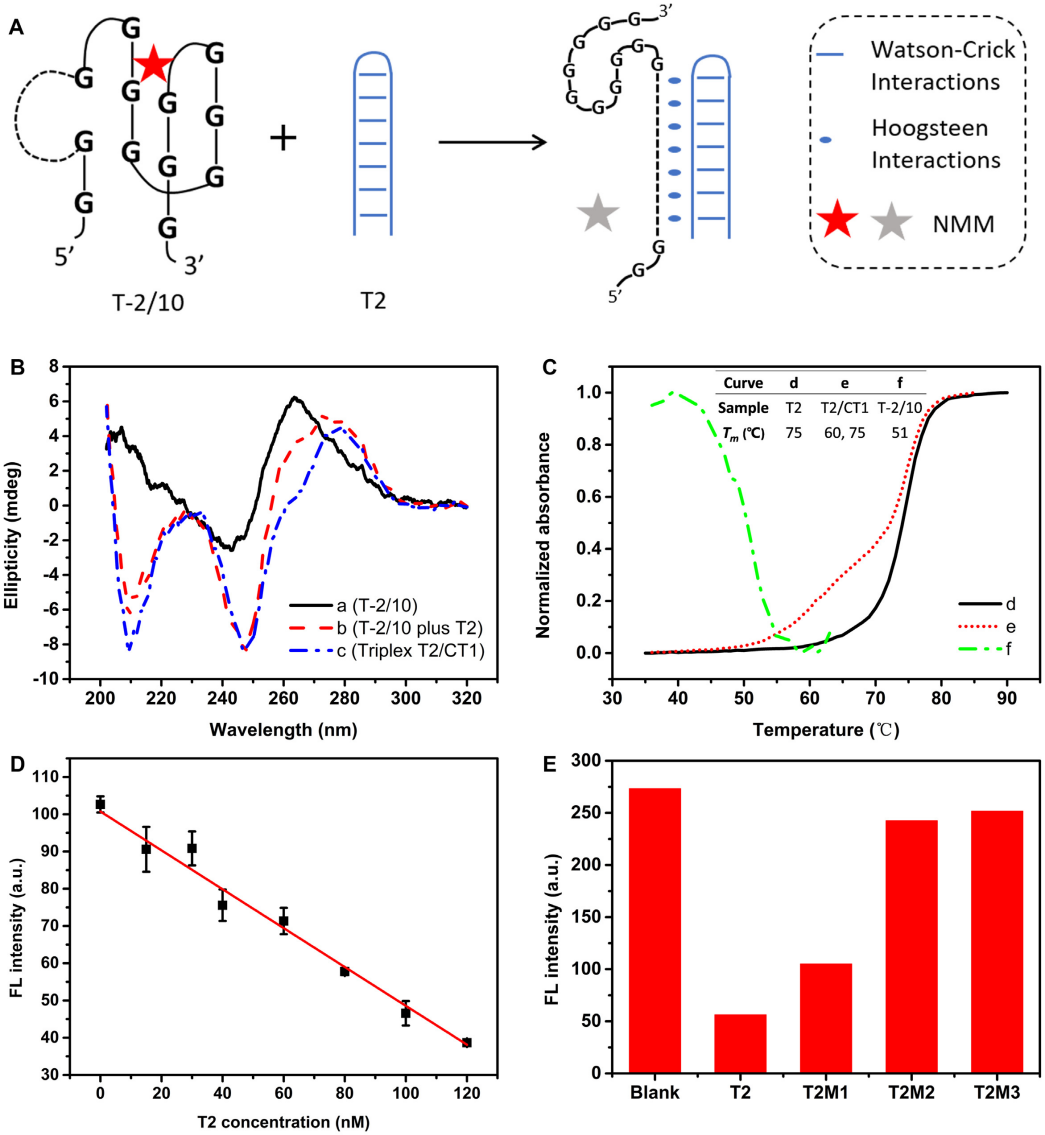
(**A**) Schematic representation of duplex (T2) detection with Intra-SG (T-2/10)-clamped molecular beacon and G-quadruplex-specific fluorescent probes (NMM), through Hoogsteen interactions. (**B**) CD spectra confirming molecular recognition between T-2/10 and T2 target. Curve c represents a hybrid structure between T2 and CT1, as a triplex control. The concentration of each DNA strand in Tris/Mg/K buffer was 7 μM. (**C**) Denaturation curves of T2 (d) and T2/CT1 (e) at 260 nm, and T-2/10 (f) at 295 nm. The concentration of each DNA strand in Tris/Mg/K buffer was 2 μM. The inserted table shows *T*_m_ values of the three samples. (**D**) A linear calibration curve for T2 determination with T-2/10-NMM system in Tris/Mg/K buffer. The error bars indicate standard deviation of three repeated measurements for each concentration of T2. The concentration of T-2/10 was 150 nM. (**E**) Fluorescence response of the system at 608 nm toward T2 and three mutated duplexes (T2M1, T2M2 and T2M3), of which one, two and three base parings were altered, respectively. The concentration of all DNA strand was 300 nM.

Aimed at facile and low-cost genetic diagnosis, specific detection of nucleic acid has been one of the most important analytical subjects in past two decades (26). In combination with G4-specific fluorescent probe, Intra-SG-clamped molecular beacons can be utilized for non-covalent (or label-free) detection of DNA. As illustrated above, non-classical single-T capped Intra-SG structures are recommended to be utilized in nucleic acid hybridization-based systems. Herein, T-2/10 was selected, due to its highest sensitivity (Figure 4B), and single-stranded DNA (T1) could be detected selectively (Supplementary Figure S5). However, it is well-known that the natural state of most nucleic acids as genetic materials is double-stranded. Generation of single-stranded DNA is inevitable for analysis of real samples and PCR products. Therefore, methods were proposed for direct detection of double-stranded DNA (27). Among these protocols, triplex-forming oligonucleotides (TFO) have attracted wide attention, since they could bind to major groove of homopurine–homopyrimidine stretches of duplex DNA in a sequence-specific manner (28,29). And these DNA sequences are abundant in mammalian genomes and could be targeted for regulation of gene expression (30). In our system, the spacer in Intra-SG (T-2/10) was designed as a TFO, which is a homopyrimidine oligonucleotide (as CT1) and able to recognize a model homopurine–homopyrimidine duplex (T2) via formation of Hoogsteen base pairs (Figure 5A). In this proof-of concept experiment, 80% TA•T (20% CG•C^+^) parallel domains are contained, which favors triplex formation at neutral pH (31,32).

CD spectroscopy was implemented for characterizing triplex generation and Intra-SG dissociation. The spectrum of T2/CT1 (curve c in Figure 5B) shows two negative bands at 210 and 247 nm and one positive peak at 279 nm, which are attributed to DNA triplex structure (33). Upon mixing of duplex T2 with T-2/10, the three representative peaks appeared (curve b), analogous to T2/CT1, indicating triplex formation between T2 and T-2/10; meanwhile, decreased ellipticity at 264 nm demonstrated dissociation of G-quadruplex, which would trigger release of G4 ligand (NMM) and fluorescence quenching. Thermal denaturation of T2 and T2/CT1 mixture in Tris-HCl buffer containing 10 mM Mg^2+^ and 20 mM K^+^ (pH 7.0, Tris/Mg/K buffer) was monitored by UV–vis spectrometer (Figure 5C). Duplex T2 underwent a normal duplex transition at 75°C (curve d). Two transitions were observed when T2 was mixed with CT1 (curve e), one transition at 75°C coincided with T2 duplex dissociation and the other one at 60°C was ascribed to triplex dissociation. The UV-melting data further demonstrated triplex formation between CT1 and duplex T2 (34). The *T*_m_ value of T-2/10 was 51°C under same condition (curve f). Appropriate relative thermodynamic stabilities (T2/CT1 > T-2/10) ensured feasibility of duplex detection with the Intra-SG probe.

Figure 5D shows fluorescence decrease from T-2/10-NMM system after titration of T2. A linear relationship between fluorescence intensity (*F*_i_) and T2 concentration (C_T2_) was obtained from 0 to 120 nM, and the linear regression equation is *F*_i_ = 100.7 – 0.5216×C_T2_/nM (*R*^2^ = 0.993). A limit of detection was calculated as 8.8 nM at a signal-to-noise (S/N) of 3, which is comparable to previous hybridization-based methods without amplification (28,35). Additionally, sequence selectivity was evaluated with mutated duplexes (Supplementary Table S1). Fluorescence response declined obviously when the base pairs (one, two or three) were altered (Figure 5E), disclosing superior selectivity of the TFO-based non-covalent probe.

Apart from direct DNA detection, Intra-SG sequences of distinct thermodynamic stabilities are promising to be designed in various DNA reactions and for sensing diverse analytes. It has been reported that monovalent cation K^+^ is quite effective to promote G-quadruplex folding (36). On the basis of the mechanism, a number of analytical platforms have been constructed for sensing K^+^ (37–39), however, Mg^2+^-assisted amplified K^+^ detection has not been reported. Distinguished from T30695 G-quadruplex, K^+^-dependent fluorescence enhancement was oberved for all those Intra-SG strands (Supplementary Figure S1L and Figure 4), resulting from K^+^-promoted G-quadruplex formation (Supplementary Figure S1 and Figure 3). Owing to the highest response (Supplementary Figure S1L), 1/11 was chosen for monitoring K^+^, instead of T-2/10. By virtue of CD characterizaton (Supplementary Figure S6A), it was confirmed that G-quadruplex can be formed in the presence of K^+^, but not with Mg^2+^ only. Nevertheless, Mg^2+^ could further stabilize G-quadruplex, which was demonstrated by increased *T*_m_ values from 38°C to 58°C after injection of 10 mM Mg^2+^ into solution containing 1/11 plus K^+^ (Supplementary Figure S6B). The remarkable phenomenon coincides with previous studies (40). Based on the principle, amplified potassium detection was realized by the aid of Mg^2+^ (g, Supplementary Figure S6C). A linear range from 2 to 100 μM was obtained, and as low as 0.8 μM K^+^ could be detected (S/N = 3), which is much better than many studies (41,42). In the absence of Mg^2+^, no evident response was observed even when K^+^ attained as high as 100 μM (h, Supplementary Figure S6C). Selectivity of Mg^2+^-assisted sensor was also tested and none of the common metal ions (Na^+^, NH_4_^+^, Ca^2+^, Cu^2+^, Pb^2+^ etc.) caused significant response (Supplementary Figure S6D).

## CONCLUSION

In summary, distinguished from commonly studied intermolecular split G-quadruplex, intramolecular split G-quadruplex (Intra-SG) has been employed in analytical area, but not been studied systematically on the influence of spacer and split mode. In order to acquire more information about its structure and property, techniques, such as CD spectroscopy, thermodynamic denaturation and UV–vis spectroscopy, were utilized. It was found that DNA spacer inserted between the two split segments did not affect the Intra-SG assembling, but it would not be conducive to interaction between G-quadruplex and ligand, if the spacer was too small (around two bases). All Intra-SG sequences could fold into G-quadruplexes in the presence of potassium ions, but exhibited distinct thermodynamic stabilities. Non-classical Intra-SG (e.g. 2:10, 4:8 and 5:7) exhibited lower stability than classical split strands (3:9, 6:6 and 9:3), which was closely related to integrity of consecutive guanine tract. In addition, as compared to regular Intra-SG structures, Intra-SG structures with a single-thymine overhang presented reduced melting temperature, providing an effective approach to the adjustment of stability and expanding their applicable scope. In the end, Intra-SG sequences were applied for construction of non-valent fluorescent biosensors, and model targets (double-stranded DNA, potassium ion) were detected with satisfactory results. In a word, this research on Intra-SG disclosed profound rules to split G-quadruplex and will contribute to its effective application in DNA technology in the future.

## Supplementary Material

gkz749_Supplemental_Files
