# Supplementary material for: Exploration of intramolecular split G-quadruplex and its analytical applications
Source: Nucleic Acids Res. 2019 Aug 31;47(18):9502–10. doi: 10.1093/nar/gkz749 (PMC6765144; doi:10.1093/nar/gkz749)

**Supporting Information for**

**Exploration of intramolecular split G-quadruplex and its analytical applications**

Mengmeng Lv,^1, 2^ Yuchun Guo,^1, 3^ Jiangtao Ren,^1*^ and Erkang Wang^1*^

1 State Key Laboratory of Electroanalytical Chemistry, Changchun Institute of Applied Chemistry, Chinese Academy of Sciences, Changchun, Jilin, 130022, China.

E-mail: [jiangtaoren@ciac.ac.cn](mailto:jiangtaoren@ciac.ac.cn); [ekwang@ciac.ac.cn](mailto:ekwang@ciac.ac.cn)

2 University of Chinese Academy of Sciences, Beijing, 100039, China

3 College of Chemistry, Jilin University, Changchun, Jilin, 130012, China

**Table S1** DNA strands used in this study. (Note: Spacer sequences were indicated by italic letters; Mutated bases were indicated in red.)

| Oligo name | Sequence (5' to 3') |
| --- | --- |
| T30695  (S0) | GGGTGGGTGGGTGGGT |
| S2 | GGGTGGGTGG*TT*GTGGGT |
| S5 | GGGTGGGTGG*TTTTT*GTGGGT |
| S10 | GGGTGGGTGG*TTTTTTTTTT*GTGGGT |
| S20 | GGGTGGGTGG*TTTTTTTTTT TTTTTTTTTT*GTGGGT |
| S30 | GGGTGGGTGG*TTTTTTTTTT TTTTTTTTTT TTTTTTTTTT*GTGGGT |
| S50 | GGGTGGGTGG*TTTTTTTTTT TTTTTTTTTT TTTTTTTTTT TTTTTTTTTT TTTTTTTTTT*GTGGGT |
| 1/11 | G*TTCTTTTCTTTTCTTTTCTT*GGTGGGTGGGTGGGT |
| 2/10 | GG*TTCTTTTCTTTTCTTTTCTT*GTGGGTGGGTGGGT |
| 3/9 | GGGT*TTCTTTTCTTTTCTTTTCTT*GGGTGGGTGGGT |
| 4/8 | GGGTG*TTCTTTTCTTTTCTTTTCTT*GGTGGGTGGGT |
| 5/7 | GGGTGG*TTCTTTTCTTTTCTTTTCTT*GTGGGTGGGT |
| 6/6 | GGGTGGGT*TTCTTTTCTTTTCTTTTCTT*GGGTGGGT |
| 7/5 | GGGTGGGTG*TTCTTTTCTTTTCTTTTCTT*GGTGGGT |
| 8/4 | GGGTGGGTGG*TTCTTTTCTTTTCTTTTCTT*GTGGGT |
| 9/3 | GGGTGGGTGGGT*TTCTTTTCTTTTCTTTTCTT*GGGT |
| 10/2 | GGGTGGGTGGGTG*TTCTTTTCTTTTCTTTTCTT*GGT |
| 11/1 | GGGTGGGTGGGTGG*TTCTTTTCTTTTCTTTTCTT*GT |
| T-1/11 | TG*TTCTTTTCTTTTCTTTTCTT*GGTGGGTGGGTGGGT |
| T-2/10 | TGG*TTCTTTTCTTTTCTTTTCTT*GTGGGTGGGTGGGT |
| T-3/9 | TGGGT*TTCTTTTCTTTTCTTTTCTT*GGGTGGGTGGGT |
| T-4/8 | TGGGTG*TTCTTTTCTTTTCTTTTCTT*GGTGGGTGGGT |
| T-5/7 | TGGGTGG*TTCTTTTCTTTTCTTTTCTT*GTGGGTGGGT |
| T-6/6 | TGGGTGGGT*TTCTTTTCTTTTCTTTTCTT*GGGTGGGT |
| T-7/5 | TGGGTGGGTG*TTCTTTTCTTTTCTTTTCTT*GGTGGGT |
| T-8/4 | TGGGTGGGTGG*TTCTTTTCTTTTCTTTTCTT*GTGGGT |
| T-9/3 | TGGGTGGGTGGGT*TTCTTTTCTTTTCTTTTCTT*GGGT |
| T-10/2 | TGGGTGGGTGGGTG*TTCTTTTCTTTTCTTTTCTT*GGT |
| T-11/1 | TGGGTGGGTGGGTGG*TTCTTTTCTTTTCTTTTCTT*GT |
| T1 | AAGAAAAGAAAAGAAAAGAA |
| T1M1 | AAGAAAAGAAAA**C**AAAAGAA |
| T1M2 | AAGAAAAGAA**T**AGAAAAGAA |
| T1M3 | AAGAAA**T**GAAAAG**T**AAAGAA |
| T1M4 | AAGAAA**T**GAA**T**AG**T**AAAGAA |
| CT1 | TTCTTTTCTTTTCTTTTCTT |
| T2 | AAGAAAAGAAAAGAAAAGAATTTATTCTTTTCTTTTCTTTTCTT |
| T2M1 | AAGAAAAGAA**T**AGAAAAGAATTTATTCTTTTCT**A**TTCTTTTCTT |
| T2M2 | AAGA**T**AAGAAAAGAA**T**AGAATTTATTCT**A**TTCTTTTCTT**A**TCTT |
| T2M3 | AAGA**T**AAGAA**T**AGAA**T**AGAATTTATTCT**A**TTCT**A**TTCTT**A**TCTT |

**Scheme S1.** The other five intramolecular split G-quadruplex structures, which are analogous to split mode 5:7, 4:8, 3:9, 2:10 and 1:11 shown in Scheme 1.


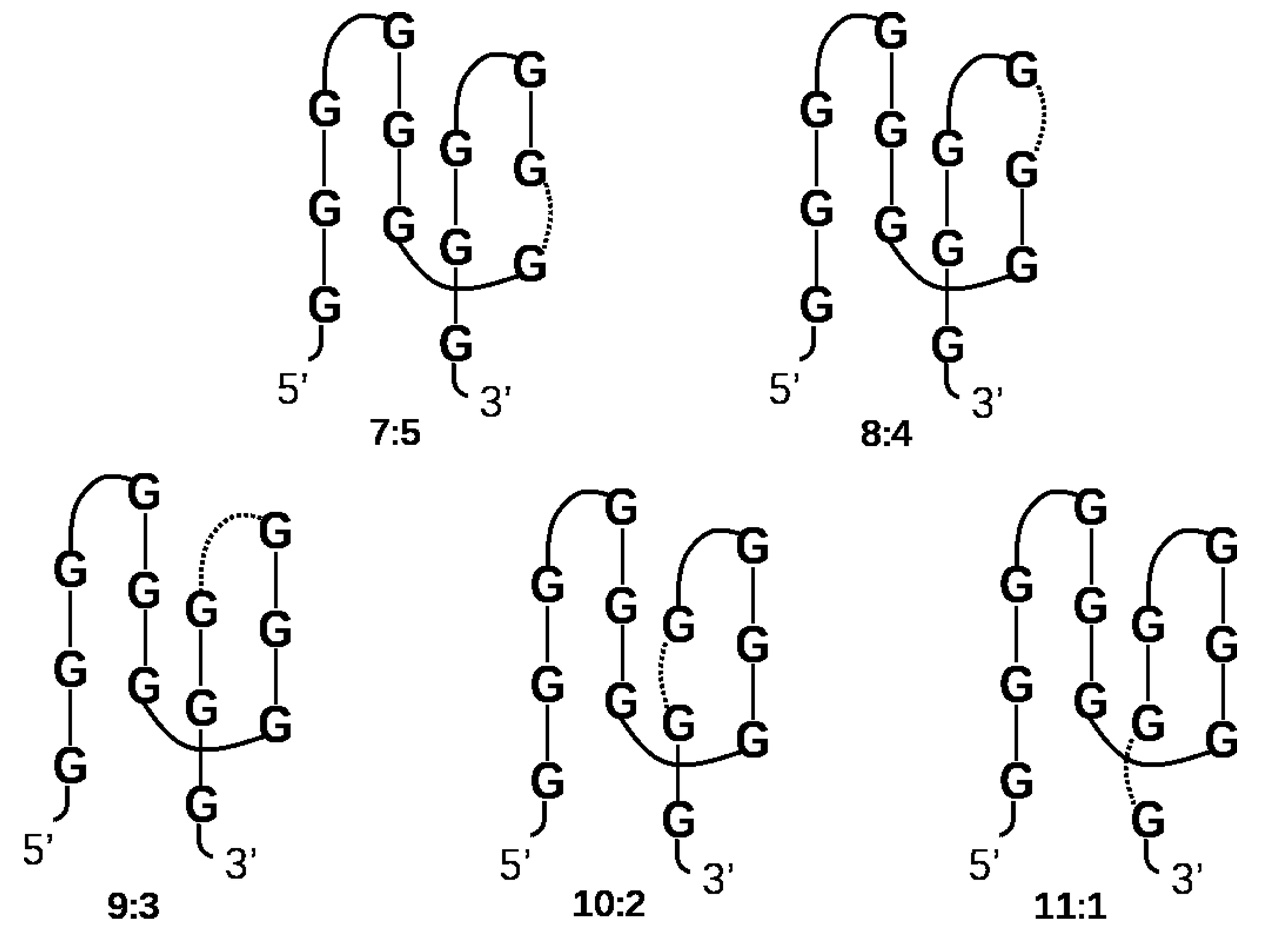


**Figure S1.** (A-K) CD spectra of Intra-SG strands in eleven split modes without or with 300 mM K^+^. The final concentration of indicated Intra-SG strand in Tris-HCl buffer (25 mM, pH 8.0) was 2 µM. (L) Fluorescence intensities of NMM in the presence of T30695 or distinct Intra-SG strands without (a) or with (b) 300 mM K^+^. The final concentrations of NMM and indicated DNA strand in Tris-HCl buffer (25 mM, pH 8.0) were 1 µM and 300 nM, respectively.


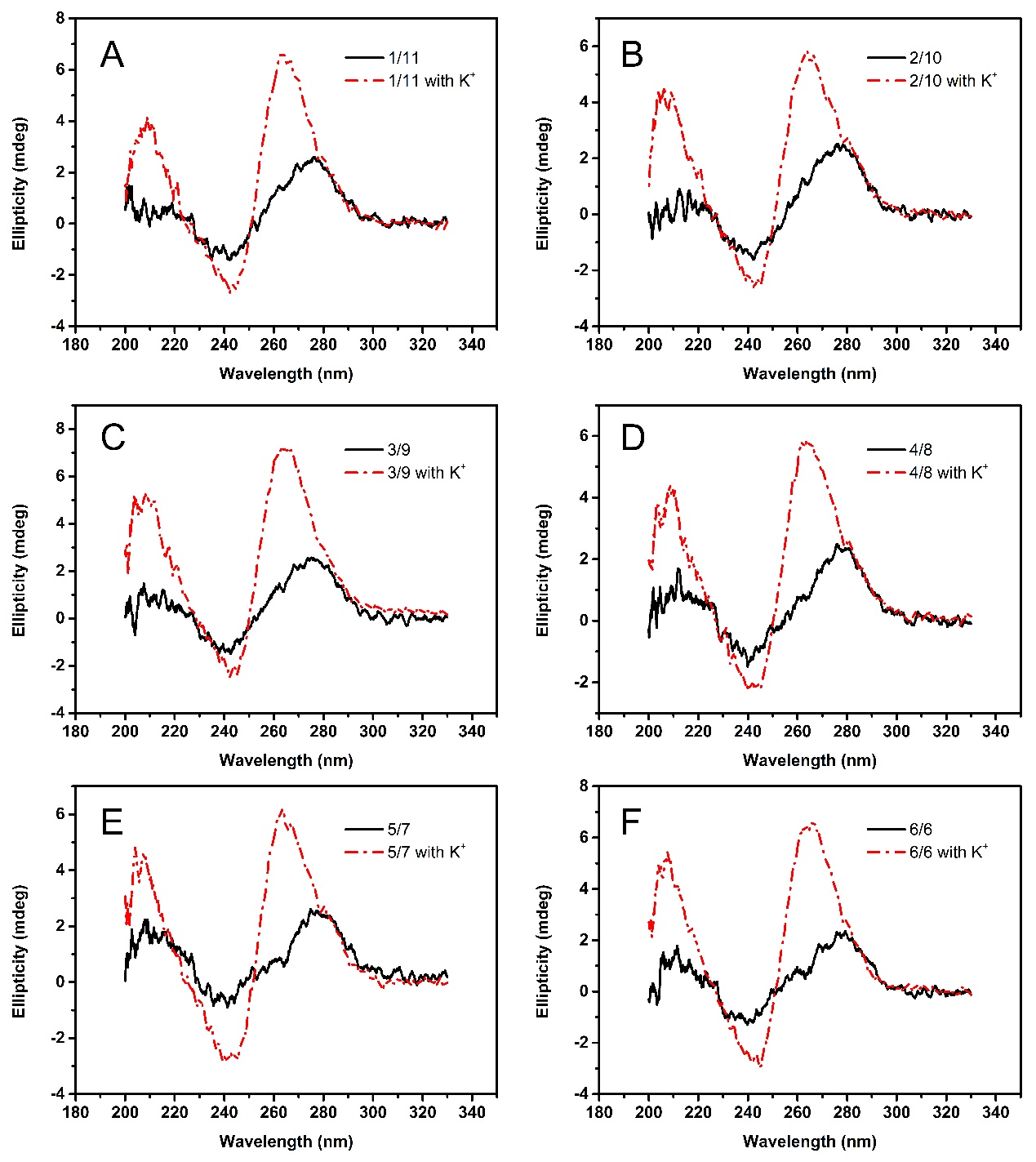


**
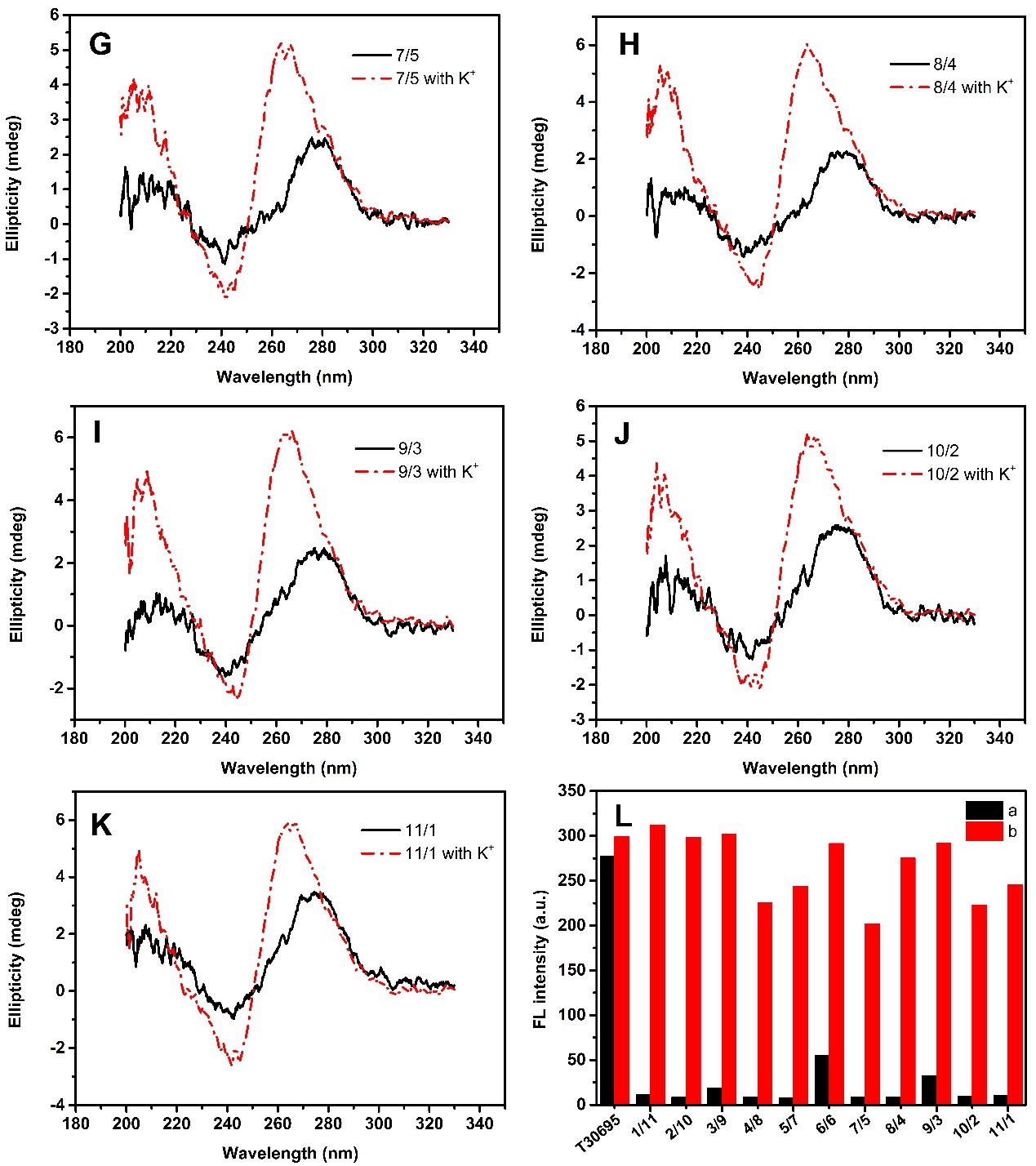
**

**Figure S2.** Native polyacrylamide gel electrophoresis (PAGE) confirming formation of intramolecular G-quadruplex from DNA strands in total eleven split modes shown in Scheme 1 and S1. All the samples (5 µl, 1 µM) and 12% polyacrylamide gels were prepared with Tirs/Mg/K buffer (25 mM Tris, 10 mM Mg^2+^, 20 mM K^+^ and pH 8.0, A), or with Tris-borate buffer (89 mM Tris and pH 8.3, B). The gels were run at 60V and 4℃, and revealed by a simple silver staining method. Migration markers are two single-stranded DNAs (M16, acaatagcttatgtaa; M36, ttctt ttctt ttctt ttctt ttctt ttctt ttcttt) and a biomolecular G-quadruplex, AGRO100 (ggtggtggtggttgtggtggtggtgg).


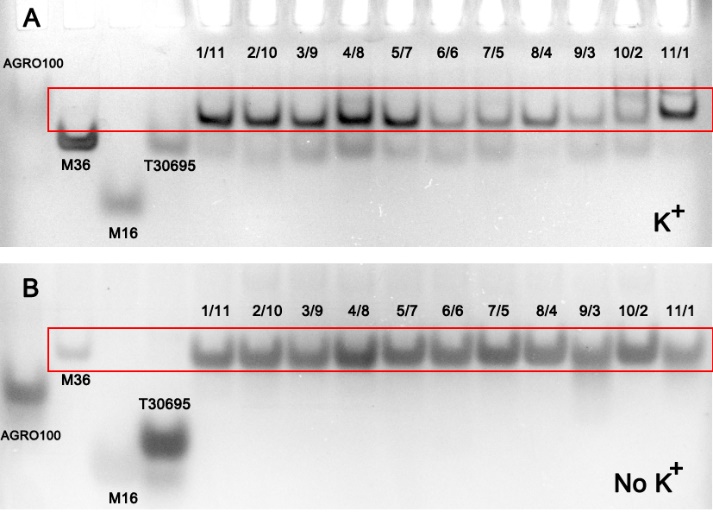


**Figure S3.** Normalized UV-melting profile of duplex T1/CT1 at 260 nm versus temperature, in the Tris-HCl buffer containing 300 mM K^+^. The obtained *T_m_* value (56℃) is also shown in Table 1. The concentration of the duplex was 2 µM.

**
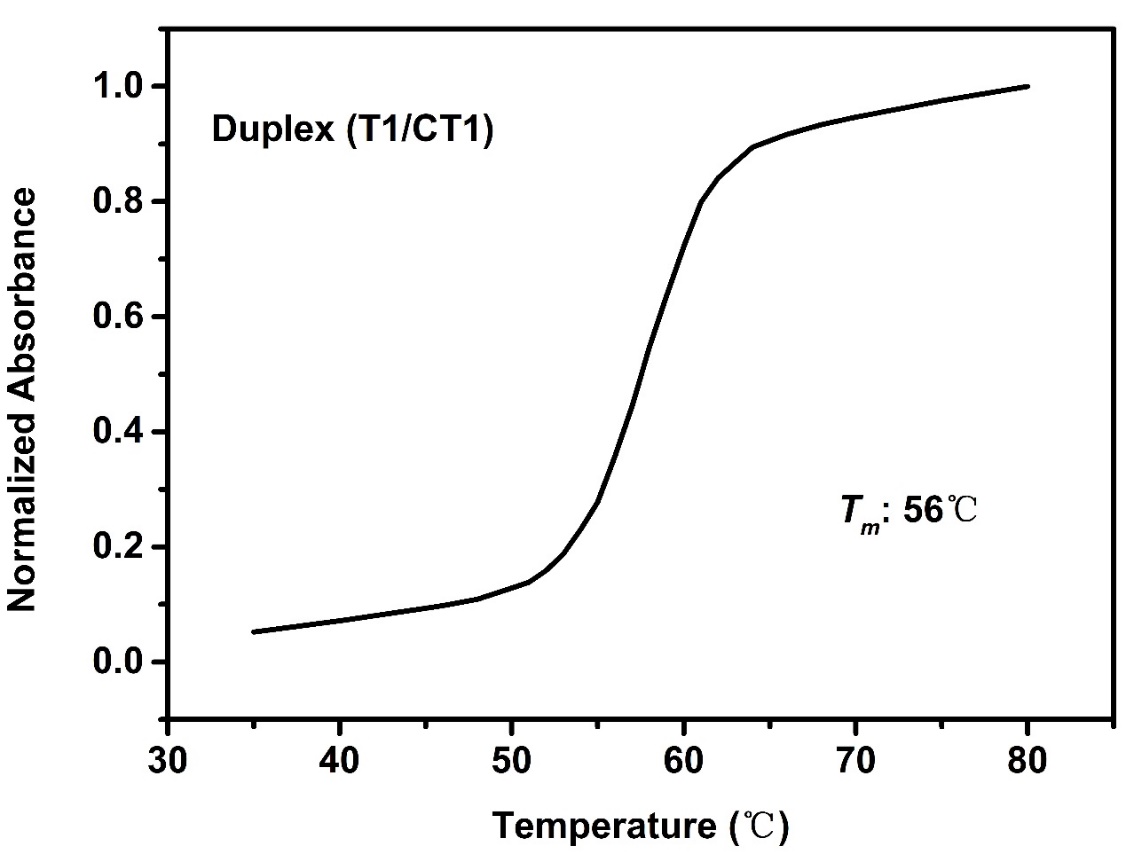
**

**Figure S4.** Normalized UV-melting profiles of regular Intra-SG strands (A) and single-T capped Intra-SG strands (B) in other five split modes shown in Scheme S1, at 295 nm versus temperature, in the presence of 300 mM K^+^. The corresponding *T_m_* values were shown in Table S2. The concentration of each DNA strand in Tris-HCl buffer (25 mM, pH 8.0) was 2 µM.


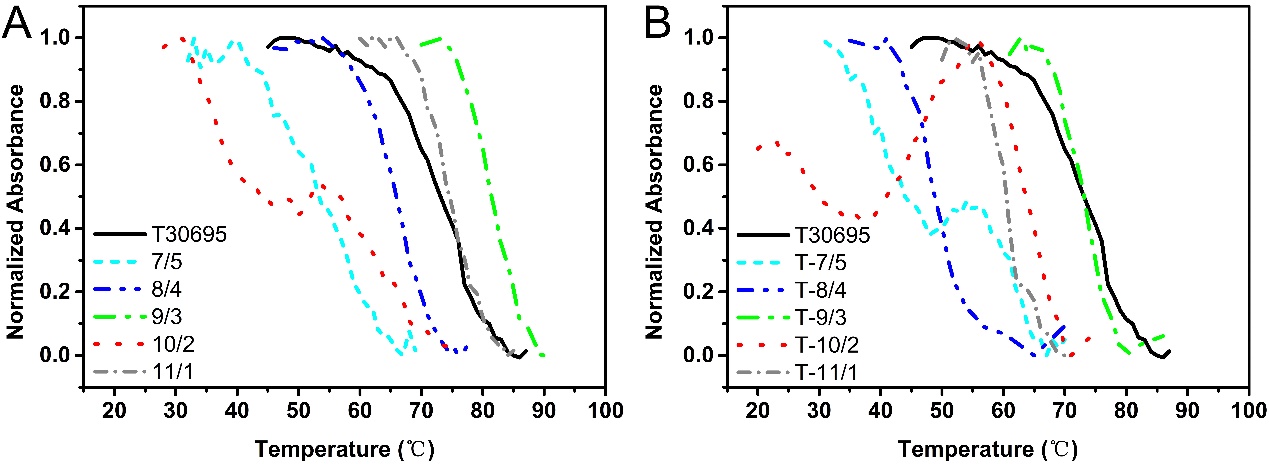


**Table S2** *T_m_* values of the other five types of Intra-SG strands shown in Scheme S1 in the presence of 300 mM K^+^, obtained by first derivative analysis of UV-melting profiles in the Figure S4.

| **Oligo** | 7/5 | 8/4 | 9/3 | 10/2 | 11/1 |
| --- | --- | --- | --- | --- | --- |
| *T_m_* (℃) | 53 | 67 | 81 | 36/67 | 73 |
| **Oligo** | T-7/5 | T-8/4 | T-9/3 | T-10/2 | T-11/1 |
| *T_m_* (℃) | 38/62 | 48 | 74 | 28/62 | 61 |

**Figure S5.** (A and B) Fluorescent spectra (A) and intensities at 608 nm (B) of T-2/10-NMM system as function of T1 concentration. A linear relationship was obtained between fluorescence intensity and T1 concentration from 20 nM to 100 nM, and a limit of detection was calculated as 15.8 nM (S/σ=3). The error bars indicate standard deviation of three independent measurements (C) Sequences of T1 and three mismatches used for selectivity investigation. (D) Fluorescent intensities of the system at 608 nm with T1 or three mutated targets. Reduced responses were revealed when mutated targets were inputted. The concentrations of NMM, T-2/10 and K^+^ in Tris-HCl buffer (25 mM, pH 8.0) were 1 µM, 150 nM and 300 mM, respectively.


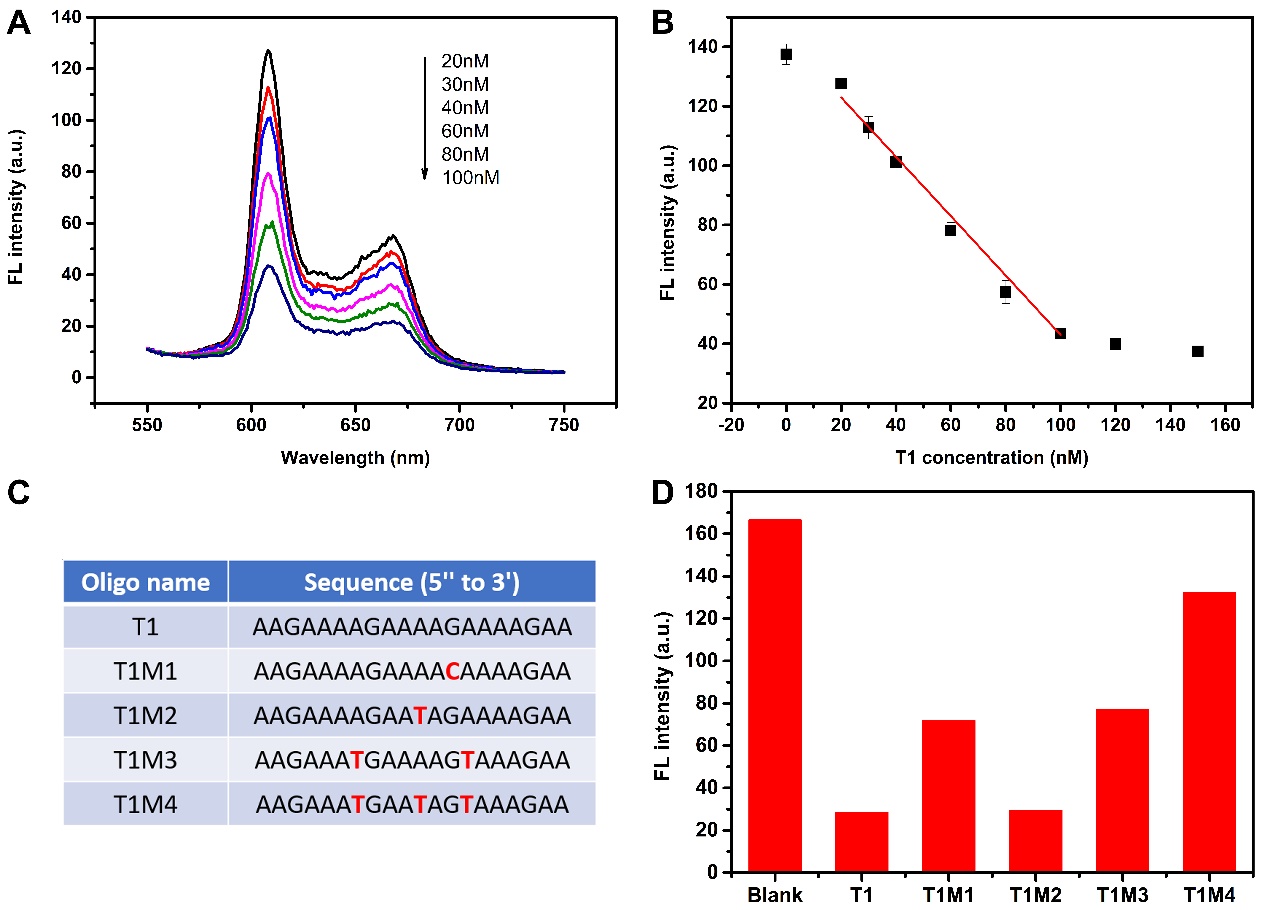


**Figure S6.** CD spectra of 1/11 without or with different metal ions (20 mM K^+^ or 10 mM Mg^2+^) in Tris-HCl buffer (25 mM, pH 8.0). The concentration of 1/11 was 7 µM. (B) UV-melting profiles of 1/11 in the presence of K^+^ without (e), or with (f) Mg^2+^ in Tris-HCl buffer (25 mM, pH 8.0). The concentration of 1/11 was 2 µM. The corresponding *T_m_* values were obtained by differentiating the profiles. (C) Fluorescent responses of 1/11-NMM system in the Tris-HCl buffer (25 mM, pH 8.0) as function of K^+^ concentration, without (g) or with (h) 10 mM Mg^2+^. (D) Fluorescent changes of the system upon addition of different ions. The concentrations of NMM and 1/11 (C and D) were 1 µM and 150 nM, respectively.


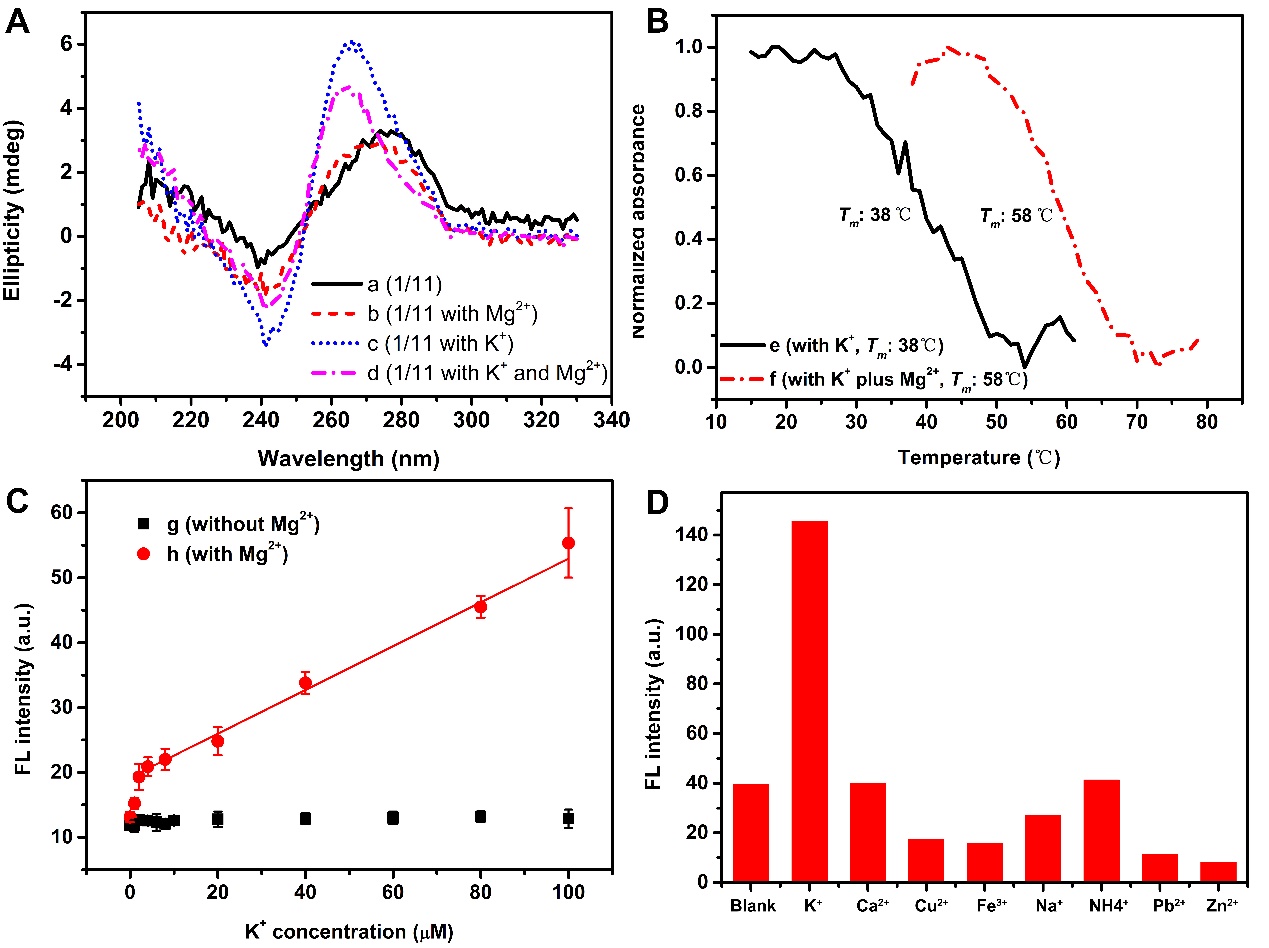

Supplement: gkz749_Supplemental_Files [file gkz749_supplemental_files.zip › Supporting Information.docx]
