# Supplementary figures and images for: Exploration of intramolecular split G-quadruplex and its analytical applications
Source: Nucleic Acids Res. 2019 Aug 31;47(18):9502–10. doi: 10.1093/nar/gkz749 (PMC6765144; doi:10.1093/nar/gkz749)

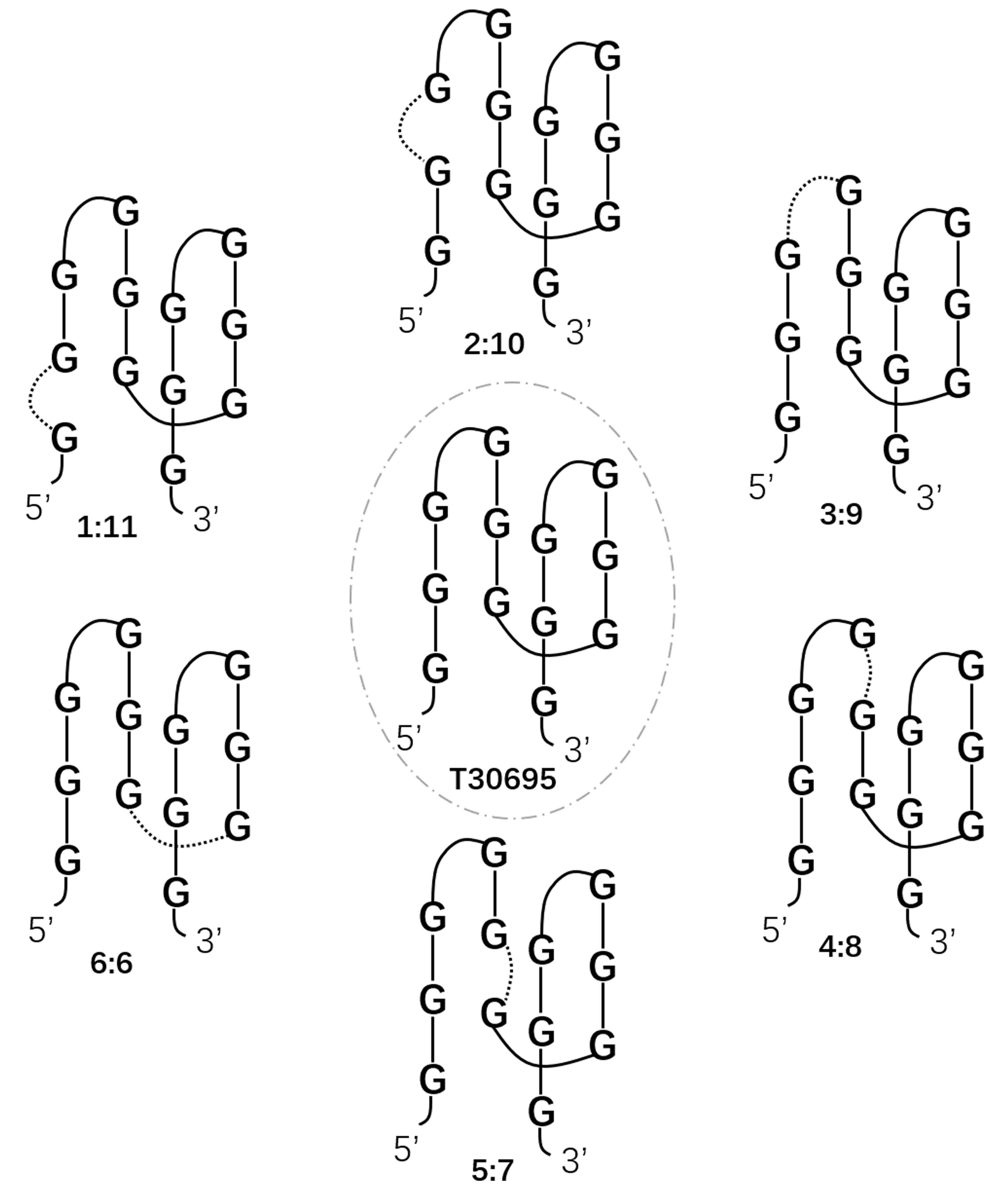

Supplement: gkz749_Supplemental_Files [file gkz749_supplemental_files.zip › Scheme 1.jpg]
